# Dwarf shrub hydraulics: two Vaccinium species (Vaccinium myrtillus, Vaccinium vitis‐idaea) of the European Alps compared

**DOI:** 10.1111/ppl.12333

**Published:** 2015-03-04

**Authors:** Andrea Ganthaler, Stefan Mayr

**Affiliations:** ^1^ Institute of BotanyUniversity of Innsbruck6020InnsbruckAustria

## Abstract

Vaccinium myrtillus and Vaccinium vitis‐idaea are two dwarf shrubs widespread in the European Alps. We studied the hydraulics of these species hypothesizing that (1) the hydraulic architecture of dwarf shrubs differs from trees, (2) hydraulic properties reflect the species' ecological amplitude and (3) hydraulic properties vary spatially and seasonally. Key hydraulic parameters (osmotic potential, turgor loss point, xylem hydraulic conductivity, vulnerability to drought‐induced embolism, stomata closure, drought‐induced cell damage and embolism repair) and related wood anatomical traits (conduit diameter and conduit wall reinforcement) were analyzed at four sites in Tyrol, Austria. Both species exhibited low hydraulic safety as well as low hydraulic efficiency. Fifty percentage embolism accumulated at −2.08 (V. myrtillus) and −1.97 MPa (V. vitis‐idaea), 88% stomata closure was at −2.19 and −2.35 MPa, respectively. After drought, both species showed embolism repair on re‐watering. Site‐specific variation within species was low, while seasonal changes in embolism resistance and turgor loss point were observed. Results indicate that studied Vaccinium species have a high risk for embolism formation. This is balanced by refilling capacities, which are probably based on the small growth height of dwarf shrubs. V. vitis‐idaea, which occurs on drier sites, showed more efficient repair and a lower turgor loss point than V. myrtillus.

**Abbreviations**
CET: Central European Time
DW: dry weight
FW: fresh weight
PLC: percent loss of conductivity
pv: pressure–volume
PSC: percent stomata closure
REL: relative electrolytic leakage
WSD: water saturation deficiency

## Introduction

*Vaccinium myrtillus* and *Vaccinium vitis‐idaea* (Ericaceae) are widespread dwarf shrubs in the European Alps (Polatschek 1999). *V. myrtillus*, the blueberry, predominantly grows in conifer forests and dwarf shrub heaths up to 2800 m. The dioecious, up to 60 cm high plant, has green, alate, multi‐branched, upright shoots, ovale light green leaves and hypogean sprouts for vegetative propagation. *V. vitis‐idaea*, the cowberry, has its main distribution in bright conifer forests, dwarf shrub heaths and mat grassland at altitudes up to 2300 m. This species is up to 30 cm high, has evergreen, leathery leaves with brown gland hair on the lower side and also hypogean sprouts. Both species prefer silicate, nutrient‐poor and humid soils. Although the species often co‐occur, *V. myrtillus* overall requires moister conditions and shady sites (Sebald et al. 1993, Polatschek 1999). *V. vitis‐idaea* can also be found on more arid and exposed sites, manifested in a higher light and a lower moisture ecological indicator value according to Landolt (2010).

Plant hydraulics are based on numerous and interrelated components and can be characterized by several key parameters: for example, the turgor loss point indicates how long a plant can maintain cell turgor on desiccation. It is determined by the osmotic concentration in cells and their wall elasticity (Lenz et al. 2006). Other important traits are the hydraulic efficiency and safety of water transport. The hydraulic efficiency depends on the hydraulic conductivity and on the ratio of supported tissue to conductive xylem area (Tyree and Zimmermann 2002). The hydraulic safety, i.e. the resistance to embolism formation, is important as the water columns in transpiring or drought‐stressed plants are under negative pressure (cohesion‐tension theory; Boehm 1893, Dixon and Joly 1894). At low water potential (Ψ), air seeding can cause embolism and, in consequence, severe dysfunctions of the transport system (Tyree and Zimmermann 2002). Most species try to avoid critical Ψ by stomata closure (Hacke and Sperry 2001, Cochard et al. 2002), but some species were also shown to be able to recover from embolism (e.g. Brodersen and McElrone 2013). A high drought resistance thus is often based on adjustments in cell osmotic potential, high resistance to embolism formation, rapid stomata reaction and xylem refilling. In addition, high cuticular resistance, an efficient root system, sufficient water storage capacity and tolerance of cells to low Ψ enable plants to withstand dry conditions.

There are countless reports on the drought resistance and hydraulics of trees (e.g. Zimmermann 1978, Tyree and Ewers 1991, Martinez‐Vilalta et al. 2004, McCulloh and Woodruff 2012), and comparably few studies dealt with the hydraulics of shrubs (e.g. Wheeler et al. 2005, Beikircher and Mayr 2008, 2009, Bucci et al. 2009, Mayr et al. 2010, Vilagrosa et al. 2010). In the database of Choat et al. (2012), xylem functional traits for 331 tree species and 140 shrubs are listed, and most shrubs represented were from arid environments. Shrubs show lower growth heights than trees and basitonic growth with annual formation of new shoots from the basal buds (Bresinsky et al. 2008). In consequence, shrubs differ from trees in transport distances, hydraulic resistances and related morphological and anatomical features (Gartner 1991, Tyree and Ewers 1991). Shrubs thus have different requirements for a safe and efficient water supply compared to trees and require different hydraulic designs. Several studies reported differences in hydraulic parameters between growth forms, for example in hydraulic efficiency (specific conductivity, leaf‐specific conductivity, Huber value), vulnerability to cavitation (drought‐induced embolism) and anatomical parameters (vessel length and diameter, cell wall reinforcement; Gartner 1991, Tyree and Ewers 1991, Patiño et al. 1995, Beikircher and Mayr 2008). Beikircher and Mayr (2008) compared shrub and tree‐like growth forms of *Juniperus communis* and found that shrub shoots were similar to tree branches in growth form and conductivity but were similar to tree stem in their vulnerability to cavitation. To our knowledge, no studies on dwarf shrubs hydraulics are available. In dwarf shrubs, the contrasting hydraulic design (compared to trees) should be even more pronounced due to their low growth height, short transport distances, small cumulative hydraulic resistances and negligible influence of gravity on water potential.

The aim of this study was a comprehensive analysis of the hydraulic architecture and drought resistance of *V. myrtillus* and *V. vitis‐idaea*. We hypothesized that (1) hydraulic key parameters and drought resistance strategies differ from trees because of the different growth pattern and lower height of dwarf shrubs, (2) interspecific differences between the species' hydraulic characteristics correspond to humidity requirements and (3) hydraulics show site‐specific and seasonal variation. We studied hydraulic parameters (osmotic potential at saturation, turgor loss point, xylem hydraulic conductivity, vulnerability to drought‐induced embolism, stomata closure and drought‐induced cell damage) and related wood anatomical characteristics (conduit diameter, hydraulic diameter and conduit wall reinforcement). Furthermore, an experiment to estimate the refilling potential of studied *Vaccinium* species was conducted.

## Materials and methods

### Sites

For sample collection and field measurements, three sites per species were chosen in Tyrol, Austria: Patscherkofel (1883 m; 47°22′N/11°47′E), Praxmar (1760 m, 47°09′N/11°07′E), Stiglreith (1368 m, 47°24′N/11°22′E; only *V. vitis‐idaea*) and Innsbruck (791 m, 47°27′N/11°37′E; only *V. myrtillus*). On all sites, widespread stands of studied dwarf shrubs can be found.

### Plant sampling

For pressure–volume (pv) curve analyses, xylem hydraulic measurements and electrolyte leakage analyses, up to 30 cm long shoots were randomly selected at each site and cut on a cloudy day after a period of rainfall to avoid high xylem tensions. Cutting was performed under water to release tension. Therefore, the basal part of the shoots was packed in a plastic bag, which was filled with water. After cutting, the shoots were kept in water and covered with a dark plastic bag, transported to the laboratory and saturated for at least 24 h, to ensure a Ψ of 0 MPa. All measurements were performed between May and October 2011. While pv curves and xylem hydraulics were analyzed for all sites, electrolyte leakage, stomata closure and anatomical parameters were measured on samples of Praxmar, due to the good accessibility of this site.

Plants from Praxmar were also used for the refilling experiment. Five soil blocks (about 40 × 40 cm, 20 cm deep) with plants of *V. myrtillus* and *V. vitis‐idaea* were extracted at Praxmar at the end of August, placed in plastic boxes and transported to the greenhouse of the Botanical Garden, University of Innsbruck.

### Water potential (Ψ)

Ψ was measured with a pressure chamber (model 1000 ‘upgraded to 100 bar’ pressure chamber; PMS Instrument, Albany, OR) on up to 10 cm long end shoots. Only in the refilling experiment, single leaves were used because of limited plant material. Therefore, the lamina was partially incised along the middle leaf vein for better sealing in the lid of the pressure chamber. It has to be noted that Ψ measurements during bench dehydration (vulnerability analyses) were suggested to reflect xylem Ψ, as stomata were closed and samples dehydrated slowly enabling sufficient equilibration of Ψ within samples. In contrast, the shoot Ψ in analysis of stomata closure probably differed from xylem Ψ due to the Ψ gradient caused by transpiration. Leaf Ψ measured in the refilling experiment can also not directly be compared with shoot Ψ as the latter integrates Ψ of several organs and tissues. Furthermore, Ψ determination on single leaves is difficult and thus less accurate.

### pv‐curve analysis (osmotic potential, turgor loss point and cell wall elasticity)

Cell osmotic parameters were analyzed via pv‐curves (Tyree and Hammel 1972), by plotting the inverse leaf Ψ (1/Ψ) vs the relative water saturation deficiency (WSD) of drying shoots. Cut and fully hydrated shoots (Ψ = 0 MPa) were dehydrated slowly on the bench, and, in intervals, Ψ and weight were determined. WSD was calculated as

(1) $$WSD=\left({FW}_{S}\;–\;{FW}_{A}\right)/\left({FW}_{S}\;–\;DW\right)\;\times \;100$$

where FW_S_ is the fresh weight of the saturated shoot, FW_A_ is the actual measured fresh weight and DW is the dry weight of the shoot. For DW determination, shoots were finally dried for 48 h at 80°C in an oven. For each shoot, 1/Ψ was plotted vs WSD (9–15 values per shoot). The turgescent section was fitted with a parabolic function and the osmotic section with a linear regression according to Boyle's law using Fig.P 2006 (Fig.P Software Inc., ON, Canada). The osmotic potential at saturation (Ψ_o_) was determined from the intersection of the linear regression with the y‐axis, the Ψ at turgor loss point (Ψ_tlp_) from the intersection of the parabolic function and the linear regression function. The cell wall elasticity (a_ela_) was assessed by the opening width of the parable. Elastic tissues show slow changes in Ψ on reduction of WSD and thus a wide parabolic function and a low a_ela_. Mean ± se for all parameters were calculated from 10 to 30 curves.

### Vulnerability to drought‐induced embolism and hydraulic conductivity

Saturated shoots [Ψ = 0 MPa, percent loss of conductivity (PLC) = 0] were dehydrated on the bench and embolism formation at different xylem Ψ determined. The main stem of shoots was immersed in distilled water and cut 2–3 cm from the basal end. Then, stem sections, up to 5 cm in length and 1–3 mm in diameter, were cut under water and decorticated. Sample ends were re‐cut several times for about 5 mm with a sharp wood carving knife and sealed in the silicone tubes of the apparatus.

To analyze the potential formation of artificial embolism by cutting the samples under tension, a control experiment with samples rehydrated for 30 min (Ψ > −0.5 MPa) prior to cutting was conducted as suggested by Wheeler et al. (2013). PLC values obtained with this method did not differ from results obtained without 30 min rehydration (data not shown), thus the formation of artifacts by cutting as well as artifacts by rapid refilling (Trifilò et al. 2014) can be excluded for the species used in our experiment.

Percent loss of hydraulic conductivity (PLC) in shoot samples was quantified by comparing the hydraulic conductivity before and after removal of xylem embolism by repeated high pressure flushes (Sperry et al. 1988). The flow rate was determined with a micro‐flow meter (µ‐Flow 500 mg h^−1^, Bronkhorst High Tech, Ruurlo, Netherlands) at a pressure of 5 kPa. Flushing (at 70 kPa; 20 min) and conductivity measurements were done with distilled, filtered (0.22 µm) and degassed water containing 0.005% (v/v) Micropur (Katadyn Products, Kemptthal, Switzerland) to prevent microbial growth. Flushing was repeated until no further increase in conductivity was observed. PLC was calculated from the ratio of initial to maximal conductivity (Sperry et al. 1988). Data (PLC vs xylem Ψ of each branch) were pooled (27–90 values) per treatment to construct vulnerability curves. Curves were fitted with an exponential sigmoidal equation according to Pammenter and Vander Willigen (1998):

(2) $$PLC=100/\left(1\;+\;\exp\left(a\;\left(\Psi \;–\;\Psi_{PLC50}\right)\right)\right)$$

where a is a constant related to the curve slope and Ψ_PLC50_ the xylem Ψ at 50% loss of conductivity. Fitting of the curves was performed with Fig.P 2006. We also calculated Ψ at 12 and 88% PLC (Ψ_PLC12_, Ψ_PLC88_).

The specific hydraulic conductivity (k_s_; m^2^ s^−1^ Pa^−1^) was measured on fully hydrated samples and calculated as

(3) $$k_{s}=Q\;\times \;l/\left(A_{c}\;\times \;\Delta P\right)$$

where Q is the maximal volume flow rate (m^3^ s^−1^), l is the length of the sample (m), A_c_ is the xylem cross‐sectional area (m^2^; calculated from the sample diameter) and ΔP is the pressure difference between the segment ends (Pa). Calculations were corrected to 20°C to account for changes in fluid viscosity with temperature. Saturated twigs always showed a PLC near zero, confirming that sample preparation did not induce artificial embolism and k_s_ values thus represent maximum conductivity.

### Stomata closure

The stomatal conductance (g_s_) was measured according to Nolf et al. (2013) on a sunny day with a SC‐1 Leaf Porometer (Decagon Devices, Pullman, WA). To determine stomata closure, g_s_ was measured on leaves of previously watered plants with maximally opened stomata (between 10:00 and 12:00 Central European Time CET). Shoots were cut and re‐positioned in the stand (similar to original position). After different periods (10 s to 2 h), g_s_ was measured a second time on the same leaves, before shoot Ψ was determined. The percent stomata closure (PSC) was calculated from the ratio of the first to the second g_s_ value and plotted vs respective shoot Ψ. Data (PSC vs Ψ) of all shoots were pooled (24–34 values) per treatment for construction of g_s_ curves. Curves were fitted according to Eqn 2 with PLC substituted by PSC and Ψ_PLC50_ corresponding to Ψ at 50% g_s_ (Ψ_SC50_). Stomata closure was defined as Ψ at 12% of maximum g_s_ (Ψ_SC12_). As stomata closure occurred within 2 h, it can be assumed that during dehydration neither light, CO_2_ concentration nor temperature or vapor pressure deficit changed considerably and stomata closure thus occurred in response to decreasing Ψ.

### Electrolyte leakage

To determine the critical Ψ for emerging cell damage, the electrolyte leakage method was used, which analyses electrolyte leakage from the symplast to the apoplast due to cell damage. By measuring the electrical conductivity of a solution with plant samples, this leakage can be assessed, whereby the conductivity of autoclaved samples (in which all cells have been killed) enables estimating the total amount of tissue electrolytes. The comparison of conductivities of dehydrated leaves to the total conductivity provides an estimation of injuries (Leopold et al. 1981, Bajji et al. 2001). Fully hydrated shoots (Ψ = 0 MPa, PLC = 0) were dehydrated on the bench. At different shoot Ψ, round leaf segments (4 mm in diameter, about 10 per sample) were cut with a cork borer, placed in test tubes with 15 ml of distilled water and shaken for 24 h at 5°C on a horizontal gravity shaker (ST5, CAT, Germany). The electrolytic conductivity (C_1_) was measured at room temperature with a conductivity meter (WTW inoLab, Weilheim, Germany). Samples were then autoclaved at 120°C for 30 min (Tuttnauer autoclave steam sterilizer 2540 ELV, Syntec GmbH Wettenberg, Germany) and shaken again for 24 h at 5°C. Afterwards, the second conductivity measurement (C_2_) was conducted at room temperature. Electrolyte leakage caused by the cutting out of the leaf segments (C_0_) was determined by measuring the conductivity of full hydrated samples and had to be subtracted from C_1_ and C_2_. Relative electrolytic leakage (REL) was thus calculated as

(4) $$REL=\left(C_{1}\;–\;C_{0}\right)/\left(C_{2}\;–\;C_{0}\right)\;\times \;100$$

Data (REL vs Ψ of each branch) of treatments were pooled (35–36 values) for construction of the curves. Curves were fitted with a sigmoid curve according to Eqn 2 with PLC substituted by REL and Ψ_PLC50_ corresponding to Ψ at 50% cell damage (Ψ_EL50_). In both species, maximum leakage on dehydration was lower than leakage on autoclaving. In dehydrating samples, cell contents were probably partly trapped within the tissue. The end points of fitted curves were therefore defined as 100% and all values were corrected in relation to the end point.

### Wood characteristics

Shoots previously used for vulnerability measurements were soaked in ethanol/glycerol/water solution (1:1:1, v/v/v) for at least 2 weeks. Cross sections (8 µm) of 10 shoots per species were cut with a microtome (Schlittenmikrotom G.S.L. 1, Schenkung Dapples, Zürich, Switzerland), stained with Etzold solution (fuchsin‐safranine‐astrablue) and analyzed with a light microscope (Olympus BX41; Olympus Austria, Wien, Austria) interfaced with a digital camera (ProgRes CT3, Jenoptik, Jena, Germany). In randomly selected radial sectors including all growth rings, areas of all conduits (68–174 per sample) were analyzed with image analysis software (ImageJ 1.45; public domain, National Institutes of Health, MD, USA). The diameters were calculated from conduit areas assuming a circular shape, averaged per sample and the mean diameter (d) per species calculated from these values. The average hydraulic conduit diameter (d_h_) was calculated from the diameter of all conduits analyzed according to Sperry and Hacke (2004):

(5) $$d_{h}\;=\Sigma d^{5}/\;\Sigma d^{4}$$

To characterize conduit wall reinforcement, the wall thickness to span ratio (t/b)^2^ (Hacke et al. 2001a) was analyzed. We measured the thickness of tangential interconduit walls (t) as well as the conduit diameter (b) for conduit pairs with average diameters within d_h_ ± 1 µm (five conduit pairs per sample). The values were averaged per sample, and the mean (t/b)^2^ per species calculated from these values.

### Refilling experiment

To test the ability of the plants to recover from drought‐induced embolism, a refilling experiment with controlled dehydration and subsequent re‐watering of plants in five soil blocks was conducted. Samples desiccated in the greenhouse, and leaf Ψ and g_s_ were measured every second day at 11:00 CET (n = 5). When leaf Ψ of both species and all blocks was about −3 MPa, re‐watering was started. Every third day, the entire blocks were submersed in a water bath for 10 min to reach soil field capacity. Monitoring of leaf Ψ and g_s_ was continued, and additional measurements of PLC were performed (n = 5 per sampling date). Samples for PLC measurements were selected randomly from the blocks, cut under water and prepared as described for vulnerability curves. The experiment was continued for 26 days.

### Statistics

All values are given as mean ± se. Differences were tested (1) at an interspecific level (*V. myrtillus* vs *V. vitis‐idaea*), (2) at a site‐specific level (populations from different sites) and (3) at a time‐specific level (measurements on *V. myrtillus* during the growing season). For vulnerability, stomata closure and electrolyte leakage analyses, differences in thresholds were tested with Welch's test and based on mean ± se values calculated from curve fittings at n‐2 df. For k_s_, Ψ_o_, Ψ_tlp_, a_ela_, d, d_h_ and (t/b)^2^, differences were analyzed with the Bonferroni test (for data with homogeneity of variance, tested with the Levene test) or Tamhane test (no homogeneity of variance) after testing for Gaussian distribution with the Kolmogorov–Smirnov test. All tests (two‐tailed) were performed pairwise at a probability level of 5% using SPSS (version 18; SPSS, IL, USA).

## Results

### pv‐curve analysis

Ψ_o_ and Ψ_tlp_ were significantly more negative in *V. vitis‐idaea* (−1.61 and −1.88 MPa) than in *V. myrtillus* (−1.22 and −1.38 MPa, Table 1). a_ela_ was lower in *V. myrtillus*, indicating a higher cell wall elasticity of this species. Between sites, no difference in Ψ_o_ and a_ela_ but partly in Ψ_tlp_ was found in both species (Table 2). In *V. myrtillus,* Ψ_o_ varied from −1.08 to −1.33 MPa, Ψ_tlp_ from −1.27 to −1.55 MPa and a_ela_ from 0.015 to 0.029. *V. vitis‐idaea* showed a Ψ_o_ between −1.51 and −1.70 MPa, Ψ_tlp_ between −1.69 and −2.09 MPa and a_ela_ between 0.026 and 0.060. Repeated measurements on *V. myrtillus* during the growing season revealed a continuous decrease of Ψ_o_, Ψ_tlp_ and a_ela_ from May to October (Table 3). The overall decline in Ψ_o_ was 0.32 MPa and in Ψ_tlp_ 0.39 MPa.

**Table 1:** pv‐curve analysis and xylem hydraulic parameters of Vaccinium myrtillus and Vaccinium vitis‐idaea. Osmotic potential at saturation (Ψ_o_; MPa), turgor loss point (Ψ_tlp_; MPa), cell wall elasticity (a_ela_; dimensionless), xylem water potential at 12, 50 and 88% loss of xylem conductivity (Ψ_PLC12_, Ψ_PLC50_, Ψ_PLC88_; MPa) and specific xylem hydraulic conductivity (k_s_; 10^−4^ m^2^ s^−1^ MPa^−1^) of shoots are given. Averaged values for three sites analyzed during summer per species in Tyrol are shown. Mean ± se, different letters indicate significant differences between species, P ≤ 0.05

	*V. myrtillus*	*V. vitis‐idaea*	n
Ψ_o_	−1.22 ± 0.03^a^	−1.61 ± 0.03^b^	30
Ψ_tlp_	−1.38 ± 0.03^a^	−1.88 ± 0.04^b^	30
a_ela_	0.024 ± 0.002^a^	0.045 ± 0.004^b^	30
Ψ_PLC12_	−0.86 ± 0.19^a^	−0.79 ± 0.18^a^	88, 90
Ψ_PLC50_	−2.08 ± 0.06^a^	−1.97 ± 0.06^a^	88, 90
Ψ_PLC88_	−3.31 ± 0.07^a^	−3.15 ± 0.06^a^	88, 90
k_s_	0.90 ± 0.15^a^	0.84 ± 0.10^a^	15

**Table 2:** pv‐curve analysis and xylem hydraulic parameters of Vaccinium myrtillus and Vaccinium vitis‐idaea on the different study sites (Pk Patscherkofel, Pr Praxmar, Ibk Innsbruck, St Stiglreith). Osmotic potential at saturation (Ψ_o_; MPa), turgor loss point (Ψ_tlp_; MPa), cell wall elasticity (a_ela_; dimensionless) and xylem water potential at 12, 50 and 88% loss of xylem conductivity (Ψ_PLC12_, Ψ_PLC50_, Ψ_PLC88_; MPa) of shoots are given. Mean ± se, different letters indicate significant differences between sites, P ≤ 0.05

	*V. myrtillus*			*V. vitis‐idaea*			
	Pk	Pr	Ibk	Pk	Pr	St	n
Ψ_o_	−1.33 ± 0.03^ab^	−1.08 ± 0.05^a^	−1.19 ± 0.01^a^	−1.51 ± 0.03^bc^	−1.70 ± 0.03^c^	−1.61 ± 0.06^bc^	10
Ψ_tlp_	−1.55 ± 0.03^a^	−1.27 ± 0.06^b^	−1.34 ± 0.01^b^	−1.69 ± 0.04^a^	−2.09 ± 0.04^c^	−1.88 ± 0.06^ac^	10
a_ela_	0.015 ± 0.002^a^	0.017 ± 0.001^a^	0.029 ± 0.004^ab^	0.060 ± 0.010^b^	0.026 ± 0.004^ab^	0.052 ± 0.006^b^	10
Ψ_PLC12_	−0.84 ± 0.28^a^	−0.98 ± 0.39^a^	−0.98 ± 0.23^a^	−0.77 ± 0.28^a^	−0.90 ± 0.30^a^	−0.82 ± 0.27^a^	27–35
Ψ_PLC50_	−1.87 ± 0.08^a^	−2.31 ± 0.13^b^	−2.08 ± 0.07^ab^	−2.20 ± 0.09^b^	−1.89 ± 0.09^a^	−1.83 ± 0.09^a^	27–35
Ψ_PLC88_	−2.90 ± 0.11^ab^	−3.63 ± 0.14^c^	−3.17 ± 0.09^b^	−3.63 ± 0.10^c^	−2.87 ± 0.11^a^	−2.84 ± 0.08^a^	27–35

**Table 3:** pv‐curve analysis and xylem hydraulic parameters of Vaccinium myrtillus in May, August and October. Osmotic potential at saturation (Ψ_o_; MPa), turgor loss point (Ψ_tlp;_ MPa), cell wall elasticity (a_ela_; dimensionless), xylem water potential at 12, 50 and 88% loss of xylem conductivity (Ψ_PLC12_, Ψ_PLC50_, Ψ_PLC88_; MPa) of shoots measured in Innsbruck, Tyrol are given. Mean ± se, different letters indicate significant differences between sampling dates, P ≤ 0.05

	May	August	October	n
Ψ_o_	−1.09 ± 0.02^a^	−1.19 ± 0.01^b^	−1.41 ± 0.01 ^c^	10
Ψ_tlp_	−1.17 ± 0.02^a^	−1.34 ± 0.01^b^	−1.56 ± 0.02^c^	10
a_ela_	0.032 ± 0.003^a^	0.029 ± 0.004^a^	0.027 ± 0.004^a^	10
Ψ_PLC12_	−1.35 ± 0.40^a^	−0.98 ± 0.23^a^	−1.03 ± 0.29^a^	30–35
Ψ_PLC50_	−2.90 ± 0.07^a^	−2.08 ± 0.07^b^	−2.36 ± 0.09^c^	30–35
Ψ_PLC88_	−4.45 ± 0.26^a^	−3.17 ± 0.09^b^	−3.70 ± 0.11^c^	30–35

### Vulnerability to drought‐induced embolism and hydraulic conductivity

**Figure 1:**
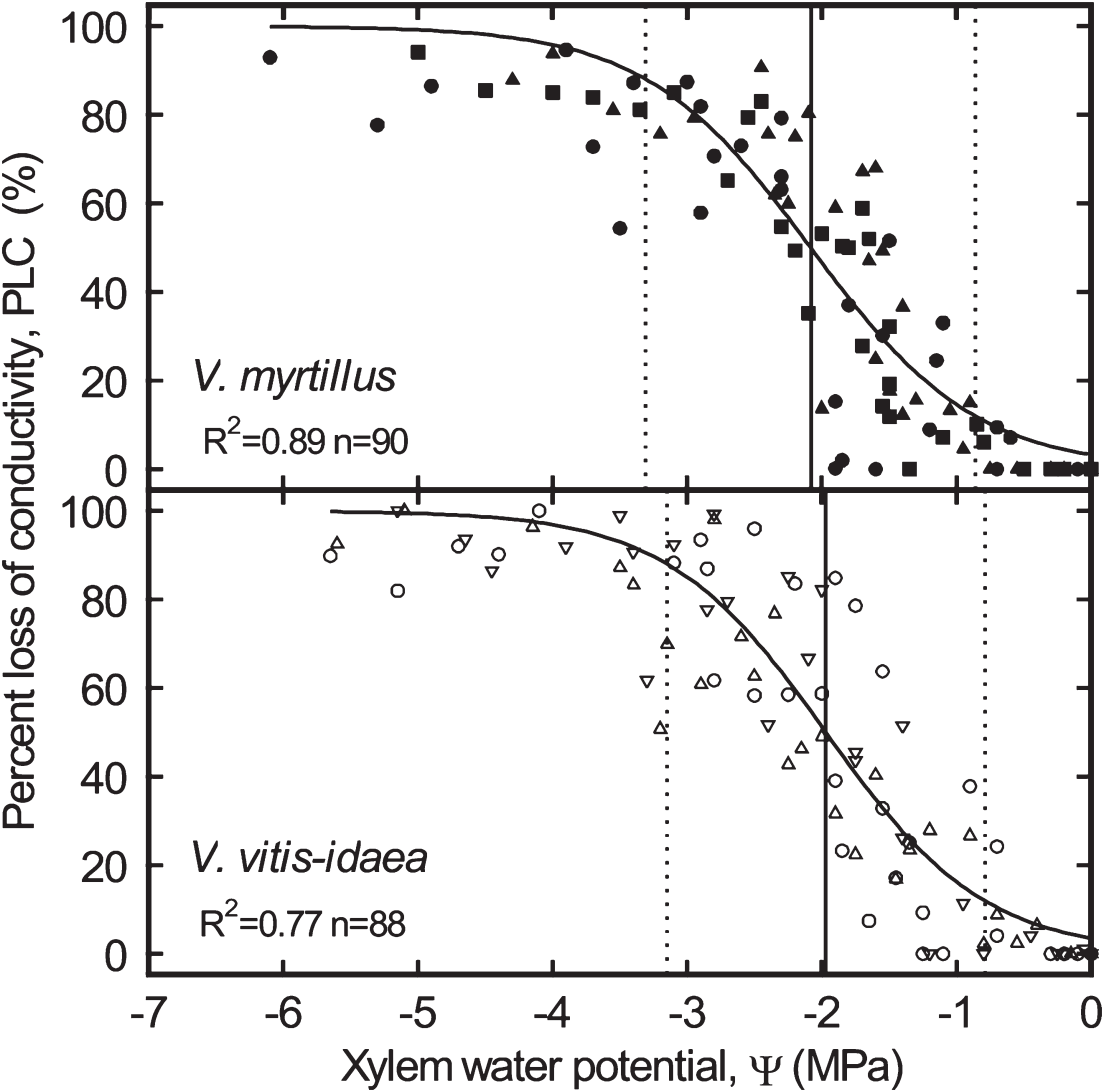
Vulnerability curves (percent loss of conductivity vs xylem water potential) of Vaccinium myrtillus (closed symbols) and Vaccinium vitis‐idaea (open symbols) measured on different sites in Tyrol: Praxmar (circles), Patscherkofel (up triangles), Innsbruck (squares) and Stiglreith (down triangles). Lines indicate water potential at 12, 50 and 88% loss of conductivity.

Both species exhibited typical sigmoid vulnerability curves with nearly identical vulnerability thresholds (Fig. 1). Embolism formation started around −0.8 MPa (Ψ_PLC12_; Table 1) and reached 50% loss of conductivity (Ψ_PLC50_) at −2.08 MPa (*V. myrtillus*) and −1.97 MPa (*V. vitis‐idaea*). Ψ_PLC88_ was at −3.31 and −3.15 MPa, respectively. Between sites, no difference in Ψ_PLC12_ was found, while Ψ_PLC50_ and Ψ_PLC88_ differed partly in both species (Table 2). Ψ_PLC50_ of *V. myrtillus* varied from −1.87 to −2.31 MPa and of *V. vitis‐idaea* from −1.83 to −2.20 MPa. The seasonal analysis of *V. myrtillus* (Table 3) revealed a noticeable decrease of the resistance to drought‐induced embolism in summer and a recovery in fall. k_s_ was slightly (not significant) higher in *V. myrtillus* than in *V. vitis‐idaea* (Table 1).

### Stomata closure

**Figure 2:**
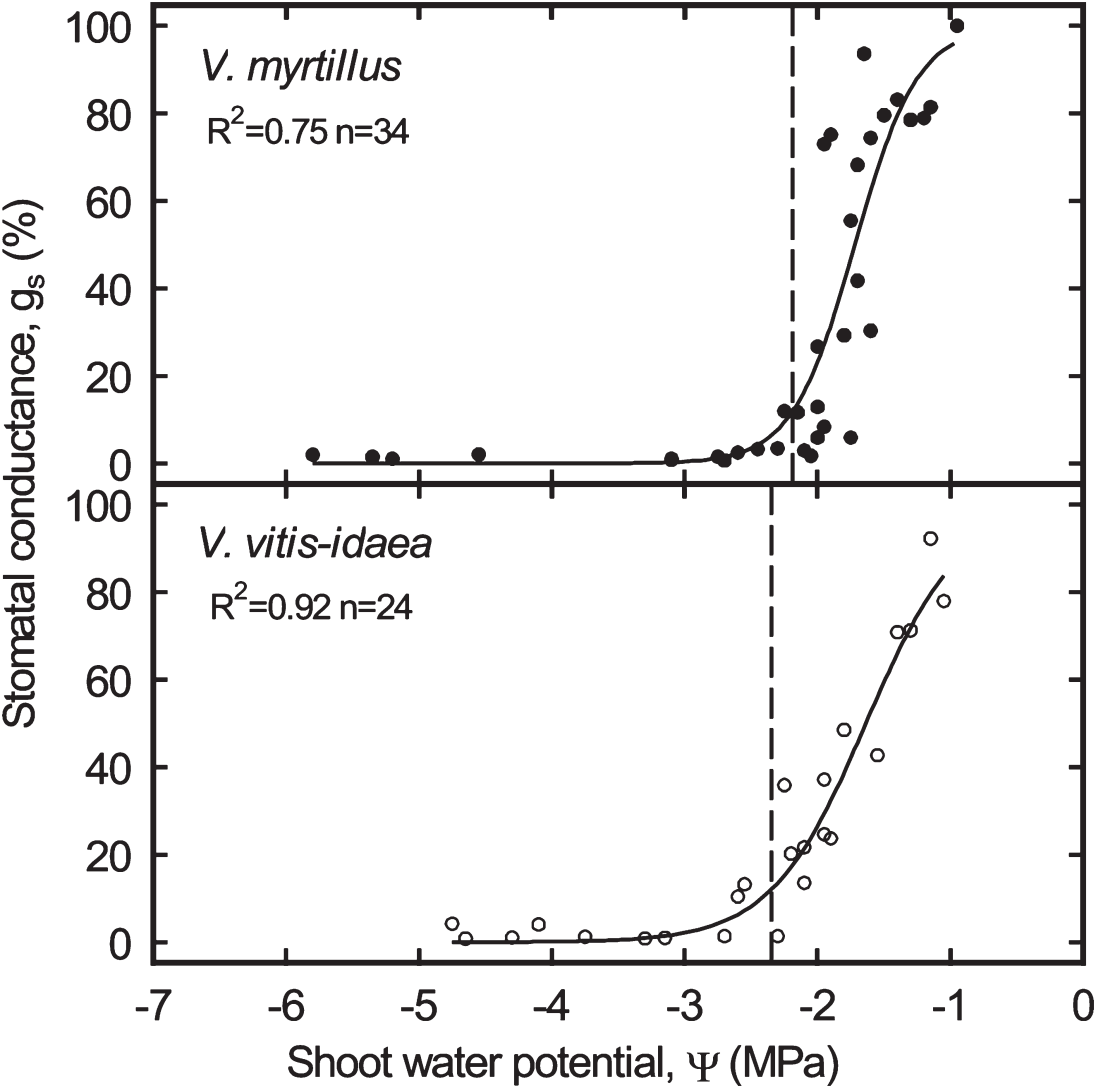
Percent stomatal conductance vs shoot water potential of Vaccinium myrtillus (closed circles) and Vaccinium vitis‐idaea (open circles) measured in Praxmar, Tyrol. Vertical lines indicate water potential at stomata closure (Ψ_SC12_).

Leaf stomatal conductance of dehydrating plants (measured on the site in Praxmar) followed sigmoid curves in both species with a slightly steeper slope in *V. myrtillus* (Fig. 2). Stomata closure (Ψ_SC12_) was observed at −2.19 MPa in *V. myrtillus* and at −2.35 MPa in *V. vitis‐idaea* (Table 4).

**Table 4:** Stomata closure of Vaccinium myrtillus and Vaccinium vitis‐idaea. Shoot water potential at 12, 50 and 88% relative stomatal conductance (Ψ_SC12_, Ψ_SC50_, Ψ_SC88_; MPa) measured in Praxmar, Tyrol are given. Mean ± se, different letters indicate significant differences between species, P ≤ 0.05

	*V. myrtillus*	*V. vitis‐idaea*	n
Ψ_SC12_	−2.19 ± 0.15 ^a^	−2.35 ± 0.12 ^a^	24, 34
Ψ_SC50_	−1.72 ± 0.05 ^a^	−1.64 ± 0.04 ^a^	24, 34
Ψ_SC88_	−1.25 ± 0.06 ^a^	−0.92 ± 0.04 ^b^	24, 34

### Electrolyte leakage

**Figure 3:**
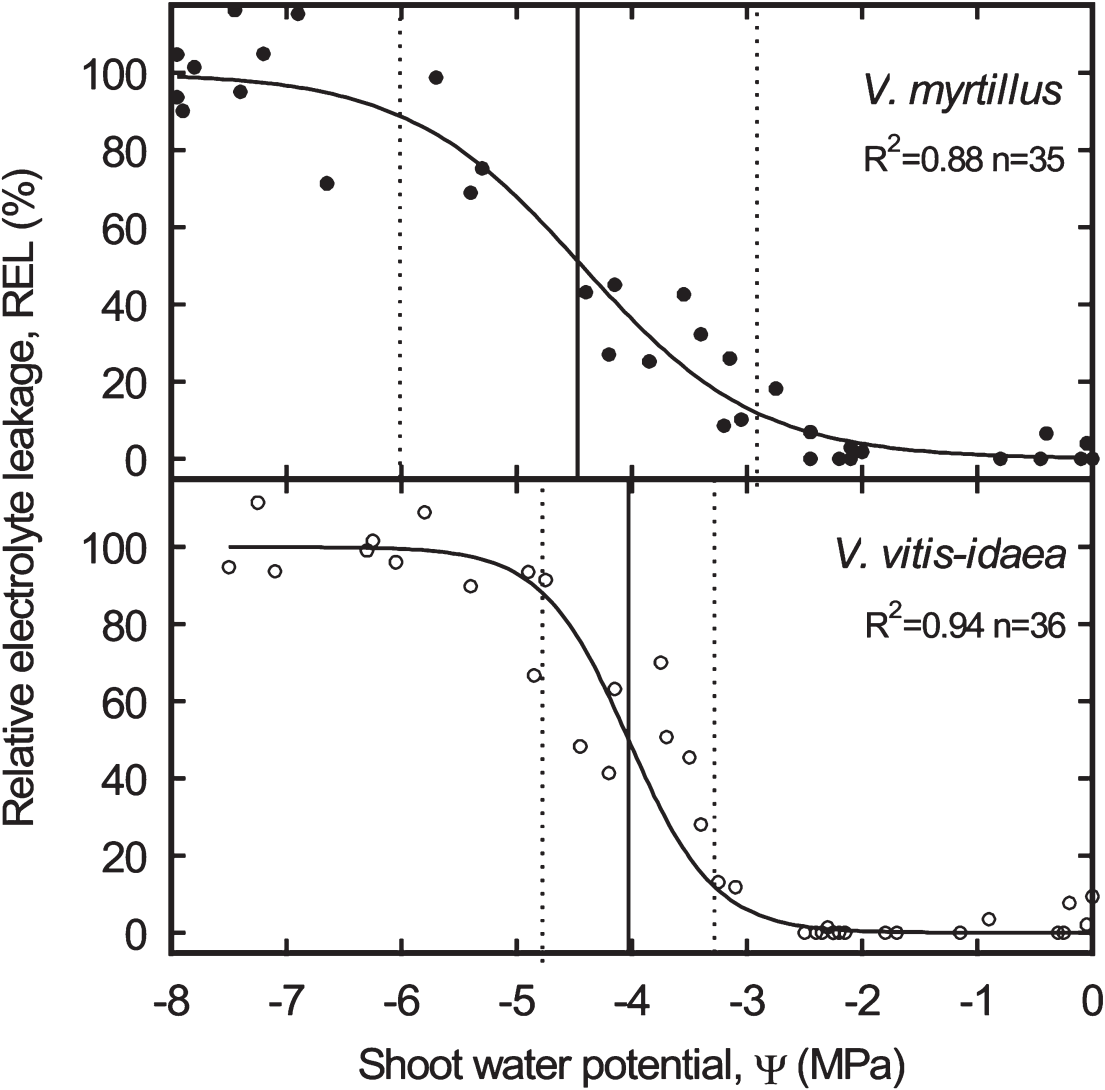
Relative electrolyte leakage vs shoot water potential of Vaccinium myrtillus (closed circles) and Vaccinium vitis‐idaea (open circles) measured on plants growing in Praxmar, Tyrol. Vertical lines indicate water potential at 12, 50 and 88% relative electrolyte leakage.

Measurements of the relative electrolyte leakage on dehydrated leaves resulted in sigmoid curves with a distinct steeper slope in *V. vitis‐idaea* (Fig. 3). A first increase in electrolyte concentration was observed in both species at about −3 MPa, but *V. vitis‐idaea* reached 50% relative electrolyte leakage at less negative shoot Ψ (−4.03 MPa) than *V. myrtillus* (−4.47 MPa; Table 5).

**Table 5:** Drought‐induced cell damage of Vaccinium myrtillus and Vaccinium vitis‐idaea. Shoot water potential at 12, 50 and 88% relative cell damage (Ψ_EL12_, Ψ_EL50_, Ψ_EL88_; MPa), analyzed with the electrolyte leakage method on plants from Praxmar, Tyrol. Mean ± se, different letters indicate significant differences between species, P ≤ 0.05

	*V. myrtillus*	*V. vitis‐idaea*	n
Ψ_EL12_	−2.91 ± 0.32^a^	−3.28 ± 0.16^a^	35, 36
Ψ_EL50_	−4.47 ± 0.12^a^	−4.03 ± 0.08^b^	35, 36
Ψ_EL88_	−6.03 ± 0.08^a^	−4.77 ± 0.01^b^	35, 36

### Wood characteristics

Both species exhibited distinct growth rings. The frequency distribution of conduit diameters showed a bell‐shaped curve for *V. vitis‐idaea*, while *V. myrtillus* contained a noticeable higher percentage of large conduits (data not shown). No significant differences in d, d_h_ or (t/b)^2^ between species were found (Table 6).

**Table 6:** Xylem anatomical parameters of Vaccinium myrtillus and Vaccinium vitis‐idaea. Mean conduit diameter (d; µm), mean hydraulic diameter (d_h_; µm) and wall thickness to span ratio [(t/b)^2^; dimensionless] of plants growing in Praxmar, Tyrol are given. Mean ± se, letters indicate that no significant differences between species were found, P ≤ 0.05

	*V. myrtillus*	*V. vitis‐idaea*	n
d	12.41 ± 0.04^a^	12.38 ± 0.07^a^	10
d_h_	16.25 ± 0.12^a^	15.67 ± 0.09^a^	10
(t/b)^2^	0.0218 ± 0.0002^a^	0.0211 ± 0.0002^a^	10

### Refilling

**Figure 4:**
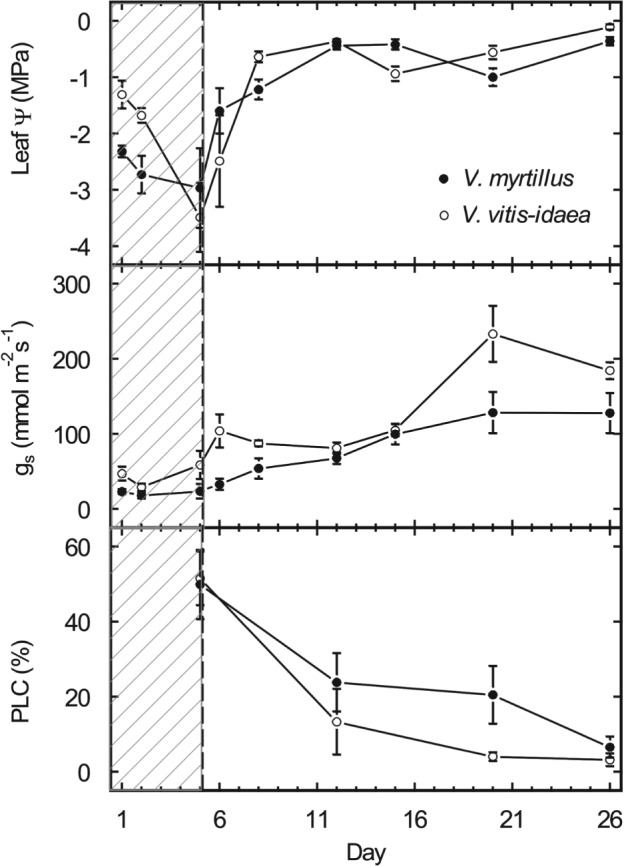
Refilling experiment with Vaccinium myrtillus (closed circles) and Vaccinium vitis‐idaea (open circles). Plants were dehydrated for 5 days until 50% loss of conductivity was reached (hatched background). Then, plants were watered periodically during 3 weeks. Leaf water potential (Ψ, MPa), Stomatal conductance (g_s_, mmol m^−2^ s^−1^) and percent loss of conductivity (PLC, %) during the dehydration and re‐watering period are given. Mean ± se, n = 5.

Fig. 4 shows the development of leaf Ψ, g_s_ and PLC of *V. myrtillus* and *V. vitis‐idaea* during 5 days of dehydration and the following 3 weeks of periodic watering. With the beginning of sufficient water supply, both species showed an increase in leaf Ψ and g_s_ and a decrease in PLC, indicating refilling of embolized conduits. *V. vitis‐idaea* revealed a distinct earlier and more pronounced recovery of g_s_ and hydraulic conductivity, with a decrease in PLC from about 50 to 13.29% within 1 week. In *V. myrtillus*, PLC decreased in the first week to 23.78%. After 3 weeks, PLC was nearly zero in both species.

## Discussion

### Hydraulic architecture of dwarf shrubs

One important aspect of a plant species' strategy in the face of limited water supply is the maintenance of cell turgor. As mean Ψ_tlp_ is −2.17 ± 0.07 MPa in temperate angiosperms (Bannister 1986), obtained results (−1.38 and −1.88 MPa, see Table 1) indicate turgor loss at relatively moderate Ψ in analyzed *Vaccinium* species. Turgor loss is known to correspond with inhibition of photosynthesis, growth and other important cell functions (Kramer and Boyer 1995). It is a reversible process as long as Ψ does not reach critical levels. In studied species, serious cell damages appeared at about −3 MPa and thus at distinct lower Ψ than Ψ_tlp_ (Fig. 3).

*Vaccinium* species exhibited a quite low hydraulic efficiency (k_s_ 0.94 and 1.11 10^−4^ m^2^ s^−1^ MPa^−1^) compared to other angiosperms (on average 17.0 ± 0.19 10^−4^ m^2^ s^−1^ MPa^−1^) and even gymnosperms (4.6 ± 0.05 10^−4^ m^2^ s^−1^ MPa^−1^; Maherali et al. 2004). Similar values were found in semi‐arid shrubs, such as *Anthyllis cytisoides* (0.72 ± 0.39 10^−4^ m^2^ s^−1^ MPa^−1^) or *Lycium intricatum* (0.99 ± 0.20 10^−4^ m^2^ s^−1^ MPa^−1^; Miranda et al. 2010). Low k_s_ cause steep Ψ gradients in plants, but due to short transport distances in dwarf shrubs, this effect may be of minor relevance. As d and d_h_ (Table 6) were similar to other alpine shrubs with higher k_s_ (*J. communis*, *Rhododendron ferrugineum* and *Rhododendron hirsutum*; Beikircher and Mayr 2008, Mayr et al. 2010), low conductivities in *Vaccinium* may not only be the result of small conduits but of high pit resistance and low proportion of conducting conduits in the xylem (Tyree and Zimmermann 2002) or non‐functional conduits in the older growth rings.

The resistance to drought‐induced embolism was also relatively low in studied *Vaccinium* species (Ψ_PLC50_ –2.08 and −1.97 MPa, see Table 1). In comparison, Ψ_PLC50_ of coniferous trees and shrubs of the timberline ecotone is between −3.39 (*Larix decidua*) and −3.98 MPa (*Picea abies*; Mayr et al. 2003, 2006). However, vulnerability thresholds of *V. myrtillus* and *V. vitis‐idaea* were similar to alpine *Rhododendron* shrubs (−1.95 to −3.24 MPa; Mayr et al. 2010). *Rhododendron ferrugineum* and *R. hirsutum* reduce the risk of xylem embolism via stomata closure 0.89–1.57 MPa above Ψ_PLC50_ (Mayr et al. 2010). Measurements on *Vaccinium* species indicated that dwarf shrubs follow a more risky strategy: although both species started to reduce stomata opening below −1 MPa, total stomata closure was not reached before −2 MPa in shoot Ψ (Table 4, Fig. 2). Studied species obviously do not rapidly close stomata on appearing drought stress and probably operate close to the point of embolism formation (Table 1, Fig. 1). It has to be noticed that shoot Ψ in g_s_ measurements cannot directly be compared with xylem Ψ of vulnerability analyses (see Materials and Methods) and that stomata reaction on rapidly increasing desiccation may differ from slowly emerging drought stress.

In contrast, efficient stomatal control and cavitation avoidance were observed in several Mediterranean shrubs suffering from intense drought during summer. Vilagrosa et al. (2003) reported a Ψ_PLC50_ of −4.81 MPa in *Pistacia lentiscus* and −6.96 MPa in *Quercus coccifera*. Other studies, however, revealed that some drought‐exposed shrubs from Mediterranean communities are only moderately resistant to cavitation (Miranda et al. 2010). In shrubs from arid Californian plant communities, Ψ_PLC50_ varied from −0.5 to −9.5 MPa (Jacobsen et al. 2007). The large variation in Ψ_PLC50_ indicates that a high cavitation resistance is only one of several strategies to cope with severe drought. Especially in shrubs, other morpho‐functional traits beyond xylem resistance may play an important role for survival.

But how can *Vaccinium* species survive in their natural habitat when they risk to suffer from embolism? The refilling experiment revealed that *V. myrtillus* and *V. vitis‐idaea* possess sufficient recovery mechanisms, which enable compensating the drawback of low hydraulic safety. Both species could remove severe embolization within 3 weeks after having achieved a balanced Ψ (Fig. 4). The increase in Ψ occurred during the first days of the re‐watering period and corresponded to restrictions in g_s_ during the first 2 weeks. An increasing number of studies document the existence and importance of refilling in different plant species (e.g. Johnson et al. 2012, Ogasa et al. 2013) and propose several underlying mechanisms, including positive root pressure, secretion of osmotica and phloem unloading (e.g. Zwieniecki and Holbrook 2009, Nardini et al. 2011, Brodersen and McElrone 2013). Our experiment simulated favorable conditions like repeated rainfalls and a wet soil as often observed at higher elevation. These conditions enabled xylem repair at Ψ of or near zero and thus are not necessarily related to ‘novel refilling’ (Hacke and Sperry 2003). In this context, the low growth height of dwarf shrubs gains in importance, as hydraulic distances are minor and may facilitate xylem repair by dissolution of entrapped air in the surrounding sap.

In summary, hydraulic efficiency and safety as well as restrictive stomata regulation are obviously less important in low growing shrubs than in trees because of short transport distances, provided that they exhibit a sufficient recovery potential. A higher performance in xylem recovery of cavitation‐vulnerable species was recently also detected in temperate tree species, indicating a general coordination between these two key parameters (Ogasa et al. 2013). It should also be considered that differences in biomechanics can affect wood traits of different growth forms (Martínez‐Cabrera et al. 2011), and shrubs can sacrifice single shoots and form new ones from the basal buds. Another aspect not included in the presented study but with potential impact on the hydraulic architecture is the clonal reproduction of the analyzed species. Physiological integration and water sharing among ramets between plants in moist and dry microhabitats can increase performance of single plants and is a major advantage of clonal growth (Alpert 1990, Schenk et al. 2008).

### Interspecific differences

Several hydraulic traits were nearly identical in both species, although *V. vitis‐idaea* can colonize drier sites. Ψ_PLC50_ did not differ between analyzed *Vaccinium* species, while on a global scale higher embolism resistance was observed in drought adapted plants (Choat et al. 2012). In contrast, the remarkably more negative Ψ_o_ and Ψ_tlp_ (Table 1) as well as the better refilling capacity of *V. vitis‐idaea* (Fig. 4) indicate a better drought adaptation of this species.

Consistently to most pv‐curve studies (e.g. Bannister 1986, Bartlett et al. 2012), Ψ_tlp_ of *V. myrtillus* and *V. vitis‐idaea* was mainly influenced by Ψ_o_ and hardly related to cell wall elasticity (Tables 1 and 3). Ψ_o_ and Ψ_tlp_ are generally connected with the aridity of a species' natural habitat (Lenz et al. 2006). Low Ψ_o_ allows a longer water uptake under soil dehydration and with low Ψ_tlp_, cells can maintain turgescence and physiological functions at low Ψ (Kramer and Boyer 1995). Thus, *V. vitis‐idaea* is able to maintain growth and photosynthesis at lower Ψ than *V. myrtillus*. Moreover, the efficient recovery of *V. vitis‐idaea* may allow an earlier increase in stomatal conductance and photosynthesis after drought events (Fig. 4). This indicates that refilling is a key hydraulic strategy of studied dwarf shrubs and that the enhanced refilling capability augments the drought resistance of *V. vitis‐idaea* considerably. Interestingly, the electrolyte leakage analysis revealed a faster increase of damages in desiccating shoots of *V. vitis‐idaea*, resulting in a less negative Ψ_EL50_ than in *V. myrtillus* (Fig. 3). As damages generally occurred in both species at low Ψ, far below turgor loss and embolization, these differences in cell damage rather reflect differences in cell composition than drought resistance strategies.

Overall, the hydraulic properties and drought resistance of studied species corresponded with their habitat demands and may explain the occurrence of *V. vitis‐idaea* on more arid sites. Although other physiological characteristics and environmental factors, such as frost hardiness and presence of a long‐lasting snow cover, may also influence habitat requirements, we suppose that water relations play an important role.

### Seasonal and site‐specific variation

*V. myrtillus* exhibited seasonal variation in several hydraulic parameters. The continuous decrease of Ψ_tlp_ and Ψ_o_ (Table 3) in developing leaves caused an increasing drought resistance during the growing season. In summer, this may be important to prevent turgor loss on midday. The observed decrease in embolism resistance from spring to summer may be due to the formation of new, susceptible conduits in early summer. Furthermore, repeated cavitation–refilling cycles during summer may reduce the resistance to air seeding (Hacke et al. 2001b). Interestingly, the hydraulic safety slightly increased in autumn. We suggest that older xylem parts were taken out of function leading to a shift in vulnerability.

In comparison to the seasonal variation, site‐specific differences in pv characteristics and in safety in both species were small, but partly significant. This indicates a (though rather low) intraspecific potential of hydraulic adaptation (i.e. hydraulic plasticity; Cochard et al. 1992) to local water availability.

## Conclusion

Hydraulic analyses on *Vaccinium* species indicated a risky hydraulic strategy with low hydraulic efficiency and safety as well as stomata closure at low Ψ. The high risk of embolism formation is probably balanced by xylem repair capacities. Hydraulics thus play an important role in the ecophysiology of dwarf shrubs and help to explain species‐specific ecological amplitudes.

## Author contributions

S. M originally formulated the idea, S. M. and A. G. designed the experiments, A. G. performed the measurements, analyzed the data, performed statistical analyses and wrote the manuscript, S. M. provided editorial advice.
